# Defining a therapeutic range for regeneration of ischemic myocardium via shock waves

**DOI:** 10.1038/s41598-020-79776-z

**Published:** 2021-01-11

**Authors:** Leo Pölzl, Felix Nägele, Jakob Hirsch, Michael Graber, Daniela Lobenwein, Elke Kirchmair, Rosalie Huber, Christian Dorfmüller, Sophia Lechner, Georg Schäfer, Martin Hermann, Helga Fritsch, Ivan Tancevski, Michael Grimm, Johannes Holfeld, Can Gollmann-Tepeköylü

**Affiliations:** 1 Department of Cardiac Surgery, Medical University of Innsbruck, Innsbruck, Austria; 2 Institute of Clinical and Functional Anatomy, Medical University of Innsbruck, Innsbruck, Austria; 3 Heart Regeneration Technologies GmbH, Innsbruck, Austria; 4 Department of Pathology, Medical University of Innsbruck, Innsbruck, Austria; 5 Department of Anesthesiology, Medical University of Innsbruck, Innsbruck, Austria; 6 Department of Internal Medicine II, Infectious Diseases, Pneumology, Rheumatology, Medical University of Innsbruck, Innsbruck, Austria

**Keywords:** Molecular biology, Cardiology, Cardiovascular biology

## Abstract

Shockwave therapy (SWT) represents a promising regenerative treatment option for patients with ischemic cardiomyopathy. Although no side-effects have been described upon SWT, potential cellular damage at therapeutic energies has not been addressed so far. In this work, we aimed to define a therapeutic range for shock wave application for myocardial regeneration. We could demonstrate that SWT does not induce cellular damage beneath energy levels of 0.27 mJ/mm^2^ total flux density. Endothelial cell proliferation, angiogenic gene expression and phosphorylation of AKT and ERK are enhanced in a dose dependent manner until 0.15 mJ/mm^2^ energy flux density. SWT induces regeneration of ischemic muscle in vivo via expression of angiogenic gene expression, enhanced neovascularization and improved limb perfusion in a dose-dependent manner. Therefore, we provide evidence for a dose-dependent induction of angiogenesis after SWT, as well as the absence of cellular damage upon SWT within the therapeutic range. These data define for the first time a therapeutic range of SWT, a promising regenerative treatment option for ischemic cardiomyopathy.

## Introduction

Ischemic heart disease (IHD) is the leading cause of death in the European Union and the Western world^1,2^. Prevalence is rising constantly due to an aging population. Ischemia results in replacement of functional cardiomyocytes with non-contractile fibrotic scar tissue. Decreased cardiac output due to a remodeled left ventricle causes the deadly syndrome of heart failure^3^. Affected patients suffer from poor quality of live with obscure prognosis^4^. Repeated hospitalizations and incapacity to work contribute to the severe socio-economic burden of heart failure^4–6^.

Despite intensive research in the field, modern pharmacotherapy mainly aims at symptom control rather than regeneration of scar tissue^7^. As novel therapeutic approaches including gene and stem cell therapy remain purely experimental, there is a major need for innovative approaches for the regeneration of ischemic myocardium^8–10^.

Shock waves are specific pressure-waves which have been used for kidney stone disintegration for more than three decades in the medical field^11^. In lower energies, they exhibit potent regenerative properties. Beneficial effects on bone non-unions, chronic tendonitis (e.g. tennis-elbow) and diabetic wound healing disorders are well described and established in clinical routine^12,13^. The observed effects are mainly attributed to the induction of angiogenesis^14–16^. Our group could show recently that the mechanical stimulus of SWs cause release of (a) angiogenic growth factors from the extracellular matrix^17^ and (b) specific extracellular vesicles containing angiogenic cargo^18^. Concomitant stimulation of inflammation enhances the angiogenic response^19^ .

Over the past years, we showed a strong angiogenic effect of SWT improving left ventricular function in ischemic cardiomyopathy in small and large animal models. Treatment caused neovascularization and resulted in reduction of myocardial scar^17,20^. Thus, SWT emerged as promising treatment strategy for ischemic cardiomyopathy. As a consequence, we initiated a prospective-randomized clinical trial, the CAST (safety and efficacy of direct Cardiac Shockwave Therapy in patients with ischemic cardiomyopathy undergoing coronary artery bypass grafting) trial (ClinicalTrials.gov Identifier: NCT03859466). The trial is currently in the recruitment phase.

Despite the promising effects of SWT and their impact on the treatment of IHD, the biophysical principles behind the therapy are not well understood. Although no side-effects have been described upon SWT, potential cellular damage at angiogenic acting energy levels was never determined in studies. Dose–response studies regarding SWT are missing so far. Thus, it remains uncertain whether the underlying mechanism of angiogenesis upon shock-wave therapy is based on a “damage-repair” principle. For this purpose, we aimed to define a therapeutic range for SW application in vitro and in vivo.

## Results

### Shock wave therapy inflicts no cellular damage at therapeutic energies

**Figure 1:**
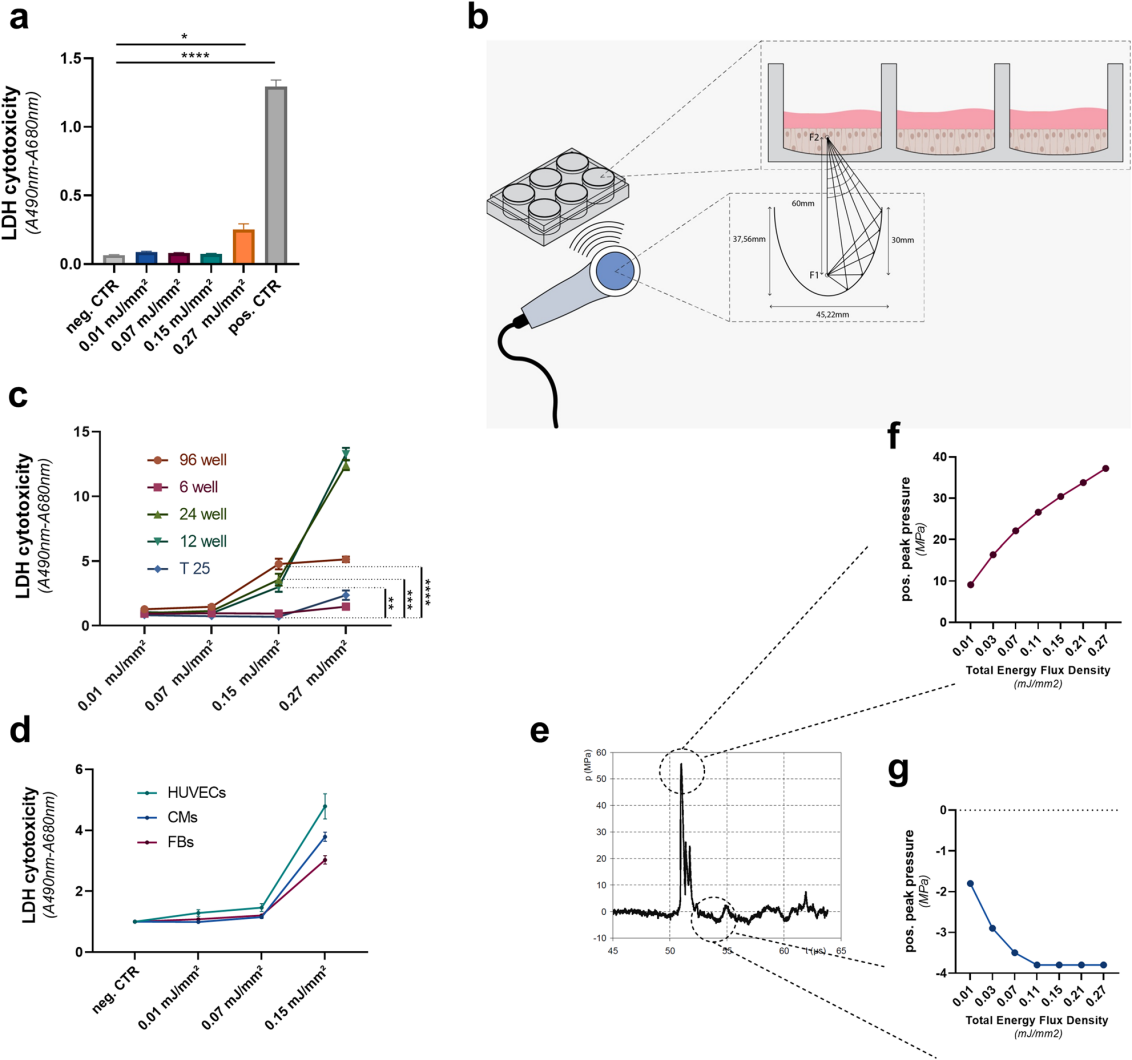
Shock wave therapy induces necrosis only at very high energy levels. (**a**) To evaluate at which energy levels SWT would induce cellular damage, we performed an LDH assay upon therapy. SWT caused no necrosis induced until energy levels of 0.27 mJ/mm^2^ total flux density in T25 flasks. Means ± SEM. *p < 0.05, ****p < 0.0001. n = 3. (**b**) Scheme for the mechanical setup of SWT in vitro: To evaluate whether the occurrence of necrosis might depend on the size and form of the cell culture flask in which the cells undergo SWT, HUVECs were seeded in 6, 12, 24 and 96 well plates and subjected to SWT. (**c**) In microwell plates with a surface smaller than 9,5cm^2^ (12, 24 and 96 well plates) necrosis could be measured at a level as low as 0.15 mJ/mm. This might be attributed to phenomena of constructive interference. Means ± SEM. **p < 0.01; ***p < 0.001, ****p < 0.0001. n = 3–4. (**d**) Comparable levels of necrosis could be discovered in fibroblasts and cardiomyocytes upon shockwave therapy. (**e**) Within the targeted tissue, shock waves induce alternating positive and negative pressure due to their specific wave profile. Positive and negative pressure induced by SWs were measured by a hydrophone showing an increase of pressure upon release of the wave with subsequent decrease to negative pressures. (**f**) Positive pressure increases concomitant with the total energy flux density and thus necrosis. (**g**) Initially, negative pressure increases concomitant with the total flux density until plateauing at a level of 3,8 MPa. Statistical comparisons between multiple groups: one-way ANOVA with Tukey post hoc analysis.

Shock waves destroy kidney stones at high energy levels, but regenerate tissue at low energy levels^11–13^.To evaluate whether SWT induces cellular necrosis at commonly used energy levels, we subjected human endothelial cells to different energy levels of SWT in T25 flasks. Release of LDH was measured 1 h after treatment to evaluate necrosis. No signs of necrosis were detected upon SWT at energy flux density levels of 0.01 mJ/mm^2^, 0.07 mJ/mm^2^ and 0.15 mJ/mm^2^ (Fig. 1a). However, we found evidence of necrosis at 0.27 mJ/mm^2^.

Shock waves are physical pressure waves, their reflection can result either in positive interference amplifying the energy of the primary wave, or cause negative interference, extinguishing the wave. Like ultrasound waves, shock waves are reflected whenever the wave encounters a material with a different density (acoustical impedance)^21^.

Thus, reflection and potential consecutive interference depends on the size and form of the cell culture flask. To evaluate whether the occurrence of necrosis might depend on the size and form of the cell culture flask in which the cells undergo SWT, we repeated the experiment using different common cell culture flasks and well plates (Fig. 1b). In contrast to the first experiment, we could observe necrosis already at a level as low as 0.15 mJ/mm^2^ in plates with a surface smaller than 9.5 cm^2^ (12, 24 and 96 well plates) (Fig. 1c). In contrast, in 6 well plates and T25 flasks necrosis only occurred at 0.27 mJ/mm^2^. These experiments suggest phenomena of constructive interference after SWT in smaller well plates. Cardiomyocytes and fibroblast inevitable receive SWT during direct epicardial application to the heart. To evaluate whether SWT induces necrosis in other cardiac domestic cells, we subjected cardio myocytes and cardiac fibroblasts to SWT. However, we could not observe any differences compared to human endothelial cells using therapeutic energy levels (Fig. 1d).

### Positive pressure induces cellular damage

Within the targeted tissue, shock waves induce alternating positive and negative pressure due to their wave profile^22^. However, whether the positive squeezing or the negative pulling acting pressure induces the described effects remains unknown.

Therefore, we evaluated pressure levels at different energy levels utilizing a hydrophone (Fig. 1e). The positive pressure increased concomitant with the total energy flux density and necrosis (Fig. 1f). The measured necrosis was thereby induced by the positive pressure of the SW, since negative peak pressure of SW treatment increased in a linear matter with corresponding energy levels, until reaching a plateau at 3.8 MPa at an energy level of 0.11 mJ/mm^2^ (Fig. 1g). For all further experiments energy levels within the therapeutiv range were used.

### SWT induces dose-dependent angiogenesis in vitro

**Figure 2:**
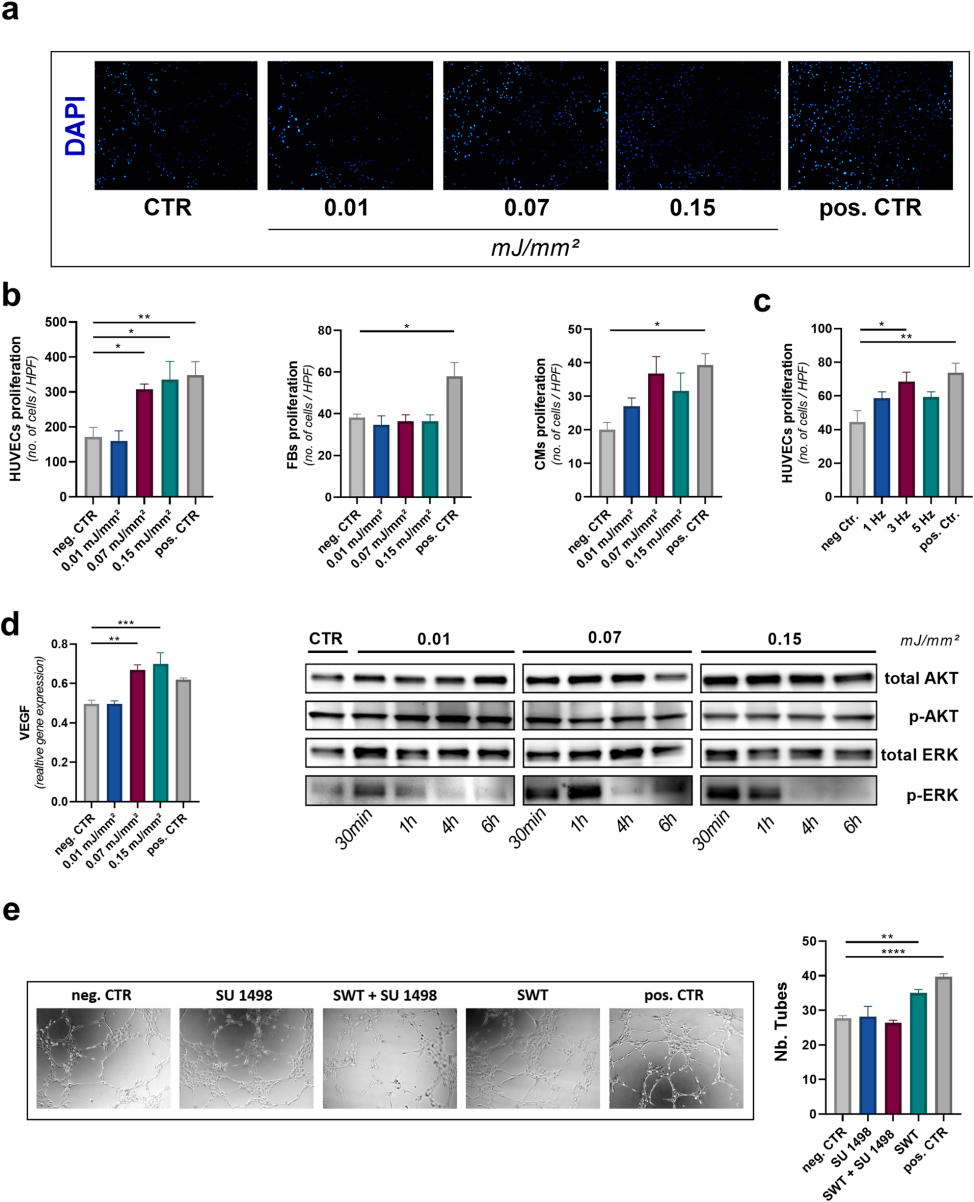
SWT induces angiogenesis in a dose-dependent manner in vitro. (**a**) To evaluate proliferation, HUVECs were subjected to SWT at different energy levels and analyzed via DAPI staining after 24 h. (**b**) SWT induced proliferation of HUVECs in a dose-dependent manner, beginning at an energy level of 0.07 mJ/mm^2^. Proliferation of cardio myocytes was enhanced upon shockwave therapy. In contrast no proliferative effect could be observed in fibroblasts. Means ± SEM. *p < 0.05, **p < 0.01. n = 6. (**c**) The highest proliferative effect of SWT was observed upon therapy at 3 Hz. (**d**) SWT enhanced the mRNA expression of VEGF in a dose dependent-manner. Means ± SEM. SWT resulted in phosphorylation of angiogenic AKT and ERK 30 min and 1 h after SW treatment. **p < 0.01; ***p < 0.001. n = 6. (**e**) To assess whether SW effects depended on VEGF signaling, a functional angiogenesis assay, the tube formation assay was performed. Angiogenic effect of SWT abolished upon treatment with VEGFR2 inhibitor SU 1498. **p < 0.01; ****p < 0.0001. n = 6. Statistical comparisons between multiple groups: one-way ANOVA with Tukey post hoc analysis. Blots are displayed in cropped format.

In a next set of experiments, we aimed to evaluate whether angiogenesis upon SWT was dose dependent. Proliferation of endothelial cells is crucial for the sprouting of newly formed vessels^23^. Therefore, human endothelial cells were subjected to SWT and analyzed for proliferation. We could demonstrate that beginning at an energy level of 0.07 mJ/mm^2^ SWT induced dose-dependent proliferation compared to untreated controls in human endothelial cells (Fig. 2a,b). In contrast, human fibroblasts showed no proliferation upon therapy. However, proliferation was enhanced after SWT in a human cardiomyocyte cell line at an energy flux density of 0.07 mJ/mm^2^ (Fig. 2b). Moreover, effects depended on frequency, as SW treatment with 3 Hz induced proliferation of endothelial cells, whereas the effect was markedly reduced with 1 or 5 Hz (Fig. 2c). In line with these findings, we found a dose dependent increase of angiogenic Vascular Endothelial Growth Factor (VEGF) expression after SW treatment (Fig. 2d). VEGF activates its receptor VEGFR2, followed by phosphorylation of the kinases AKT and ERK both of which induce angiogenesis^24,25^. To evaluate angiogenic signaling upon SWT, we analyzed phosphorylation of both AKT and ERK after SWT. SWT induced phosphorylation of AKT and ERK 30 min and 1 h after SW treatment. Interestingly, although phosphorylation of AKT was independent of SW dose, activation of ERK was dose-dependent (Fig. 2d). These findings indicate a dose dependent angiogenic and proliferative effect of SWT. To assess whether SW effects depended on VEGF signaling, we performed a tube formation assay in the presence of the VEGFR2 inhibitor SU1498. Angiogenesis upon SWT was abolished upon pretreatment with VEGFR2 indicating a crucial role of the VEGF axis in the angiogenic response upon SWT (Fig. 2e).

### SWT improves limb perfusion

**Figure 3:**
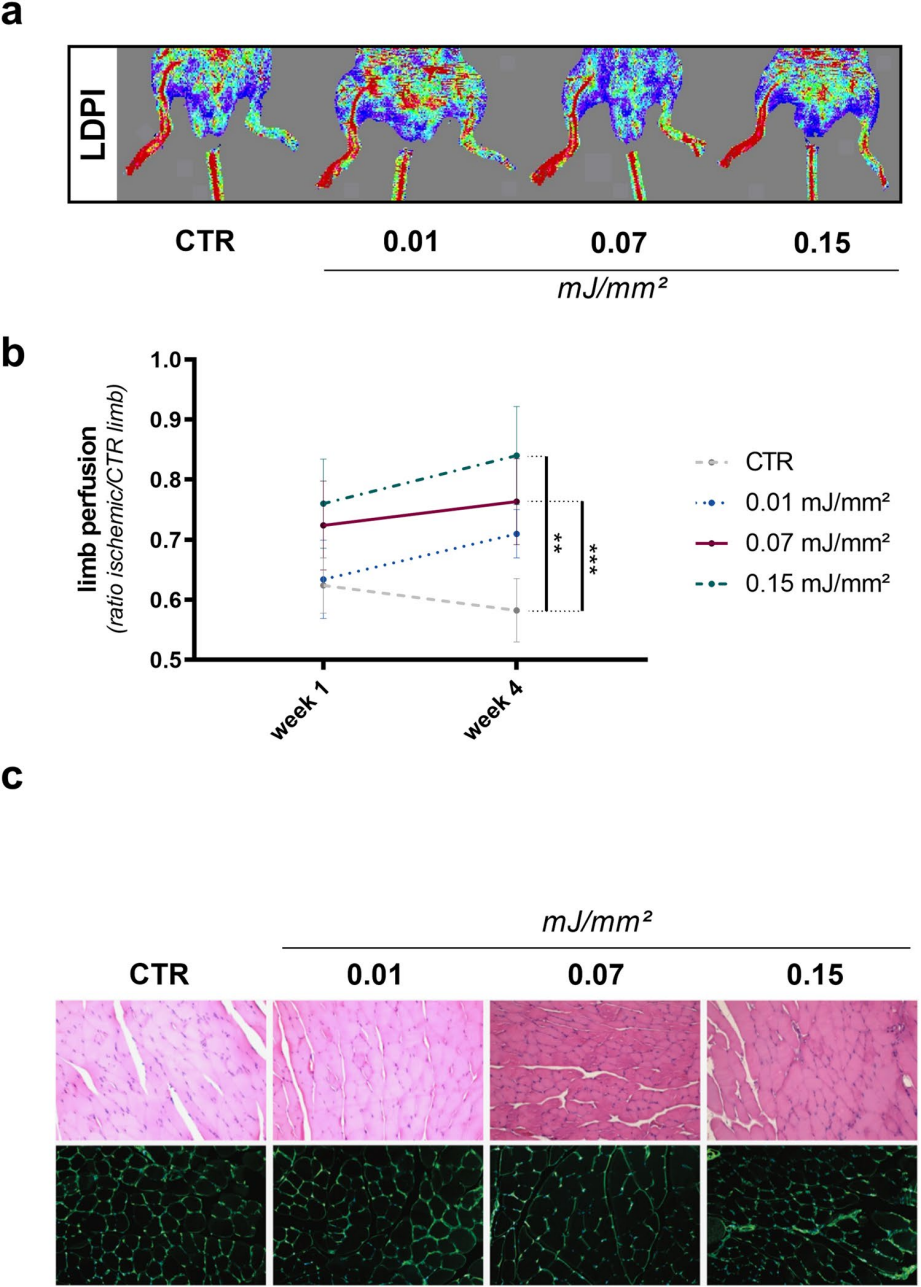
SWT improves limb perfusion dose-dependently. (**a**, **b**) To evaluate dose–response relationship in vivo, mice were subjected to hind limb ischemia and treated with different SW doses thereafter. Treatment with 0.07 and 0.15 mJ/mm^2^ improved limb perfusion 4 weeks after treatment compared to untreated controls, whereas treatment with 0.01 mJ/mm^2^ showed no beneficial effect . Means ± SEM. **p < 0.01; ***p < 0.001. n = 5–10. (**c**) To asses necrosis upon SWT in vivo, limbs of mice were treated with SWT. Upon treatment with different levels of energy flux density, no signs of necrosis could be observed in pathological examination. Statistical comparisons between multiple groups: one-way ANOVA with Tukey post hoc analysis.

To assess dose-dependency in vivo, we performed SWT in a murine model of hind limb ischemia. We found improved limb perfusion compared to untreated mice 4 weeks after SW treatment (Fig. 3a). As anticipated from our in vitro findings, energy levels of 0.07 and 0.15 mJ/mm^2^ had the most beneficial effects on hind limb regeneration (Fig. 3b). To evaluate possible cytotoxic effects in vivo, SW treated muscle samples underwent pathological examination. There was no evidence of necrosis or cellular damage, independent of dose (Fig. 3c).

### SWT induces angiogenesis in vivo

**Figure 4:**
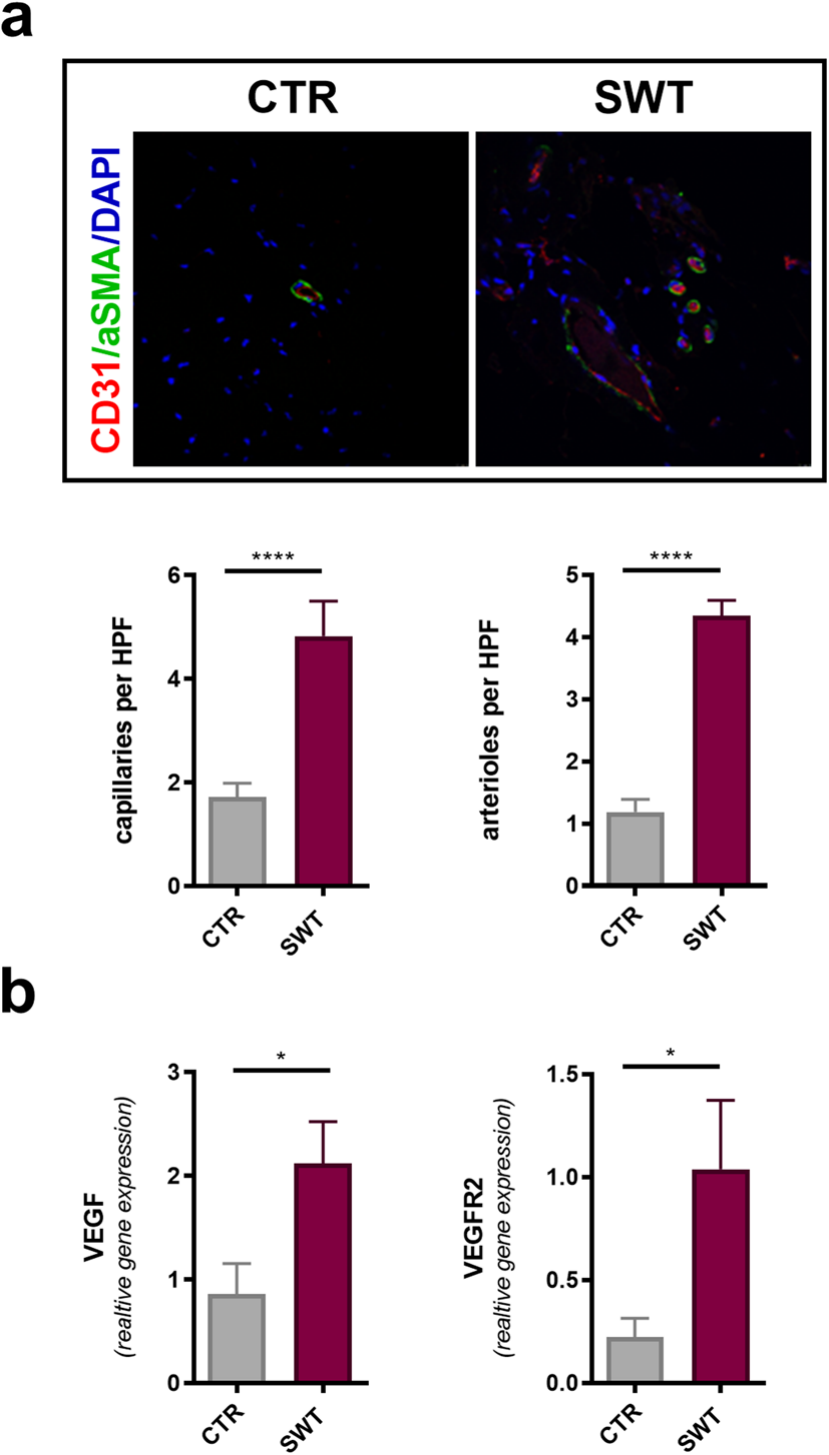
SWT induces angiogenesis in vivo. (**a**) To evaluate angiogenesis in ischemic muscles, we performed immunostaining of endothelial cells and vascular smooth muscle cells. Quantification showed increased numbers of capillaries and arterioles in ischemic limbs subjected to SWT. Means ± SEM. ****p < 0.0001. n = 5–10. (**b**) qPCR revealed increased gene expression of VEGF and VEGFR2 in ischemic muscle 3 days after SW treatment. Means ± SEM. *p < 0.05. N = 5–10. Statistical comparisons between two groups: Student’s *t* test.

To assess whether improved limb perfusion was due to angiogenesis we stained ischemic muscles for newly formed vessels. We found an increase of capillaries as well as arterioles in mice treated at an energy level of 0.07 mJ/mm^2^ in comparison to untreated animals (Fig. 4a). In parallel, gene expression levels of VEGF and its receptor VEGFR2 were increased after SWT (Fig. 4b).

## Discussion

The prevalence of ischemic heart failure rises due to constantly aging population. Frequent rehospitalizations and incapacity to work cause a severe socio-economic burden for Western health care systems^4–6^. Despite modern pharmacotherapy, ischemic heart failure is still associated with poor outcomes. Current treatments aim at symptom control rather than myocardial regeneration. Novel therapies as gene or stem cell therapy showed promising results in pre-clinical experiments. However, they have failed to gain routine clinical application due to unfavorable side effect profiles and ethical concerns^8–10^. Therefore, there is major need in novel and safe therapy for patients suffering from ischemic cardiomyopathy.

Shockwave therapy has been used in clinical routine in various indications for more than 30 years without showing any form of side effects^11^. Beneficial effects of SWT in ischemic heart failure via induction of angiogenesis were shown before^18,20,26–28^. On the other hand, SWT has been used at much higher energy levels for the destruction of kidney stones. Hence, depending on the used energy level, SW can both induce tissue regeneration as well as stone disintegration. However, the dose–response profile of SWT has not been defined so far.

In this work, we could define for the first time a therapeutic range of SWT. Within the therapeutic range, SWT did not cause cellular damage. Furthermore, we could show that necrosis beyond energy levels of 0.27 mJ/mm^2^ is attributed to positive pressure, since negative pressure released by SW plateaus already at an energy level of 0.11 mJ/mm^2^.

Other cellular responses to increased pressure upon SWT, as the formation of caveolae, have been described previously^29^. However, earlier works performed experiments only at one single level of energy flux density.

Since a therapeutic range for SWT has not been defined yet, we subject endothelial cells to SWT at different energy levels. We could demonstrate that the proliferative effect of SWT is dose dependent and plateaus at an energy level of 0.07 mJ/mm^2^. Concomitant with these findings we could observe a dose depended increase of angiogenic gene expression with subsequent phosphorylation of protein kinases AKT and ERK upon SWT. Therefore we could demonstrate in vitro an extended therapeutic range of the SWT, since experiments in earlier works were only performed at an energy flux density of 0.08 mJ/mm^2^^19,26^.

In a murine model of hind limb ischemia we investigated the effects of SW treatment on ischemic tissue. As anticipated from earlier in vitro findings, we could show again a dose dependent increase in limb perfusion in animals subjected to SWT. Immunofluorescence staining revealed an increased amount of capillaries and arterioles in ischemic muscles after SWT. Combined with increased angiogenic gene expression in vivo after SWT, these findings underline the angiogenic effect of SWT. However, these experiments show a dose–response profile in soft tissue only. Whether the beneficial effects of SWT on bone non-unions is in the same therapeutic range has to be demonstrated in future works^12,13^. We found no damage after SWT in muscle tissue, previous studies found no evidence of cellular damage after SWT of ischemic myocardium^17,20^.

**Figure 5:**
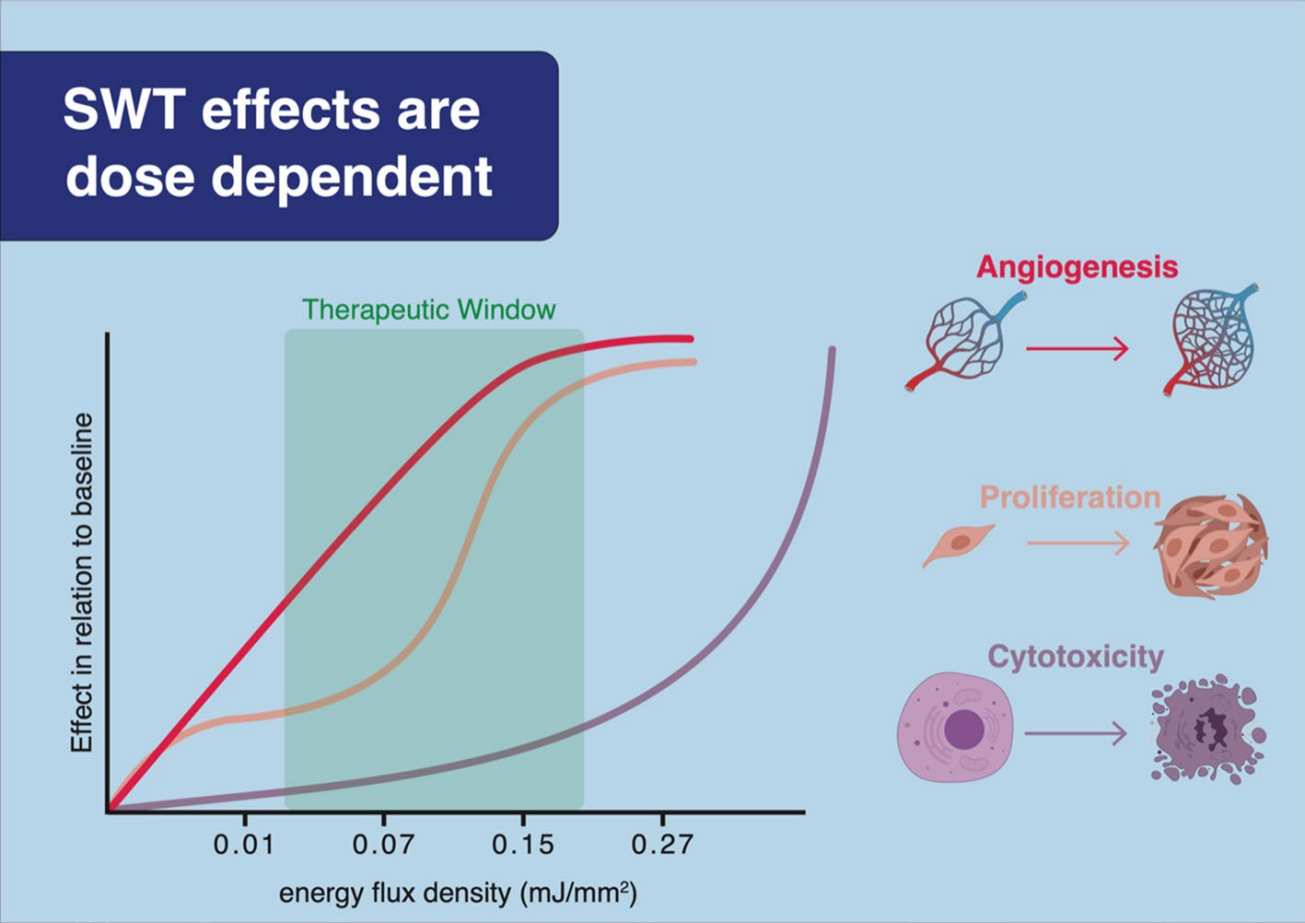
Central picture. These data define for the first time a therapeutic range of SWT. We provide evidence for a dose-dependent induction of angiogenesis after SWT, as well as the absence of cellular damage upon SWT within the therapeutic range.

Overall, we provide evidence for a dose-dependent induction of angiogenesis after SWT (Fig. 5). VEGF signaling resulted in endothelial proliferation and neovascularization improving limb perfusion of ischemic muscle. We were able to define a therapeutic range of SWT from 0.07 to 0.15 mJ/mm^2^. Moreover, we could show the absence of necrosis upon SWT within the therapeutic range. Hence, the angiogenic properties are not attributed to a “damage-repair” mechanism, but rather due to a specific induction of angiogenic signaling pathways. Combined the results of this study and earlier works, suggest the SWT as a novel and side effect free treatment option for ischemic heart failure^17,18,20^. Translating our findings to a clinical setting, the currently recruiting CAST trial (ClinicalTrials.gov Identifier: NCT03859466) investigates the safety and efficacy of cardiac SWT during CABG surgery for myocardial regeneration.

## Materials and methods

### Cell culture

Human umbilical vein endothelial cells (HUVECs) were isolated from umbilical cords obtained by Caeseream sections. Informed consent was obtained from all participants and/or their legal guardians. Approval of this study protocol was given from the ethics committee of Innsbruck Medical University (no. UN4435) and complied to the Declaration of Helsinki. Isolation of endothelial cells was performed as described in detail before^19^. HUVECs were grown in endothelial growth medium (EGM) (Lonza, Basel, Switzerland) and cultured as described previously. Fibroblasts and cardio myocytes were purchased from Promo Cell (Heidelberg, Germany) and cultured in DMEM containing 10% FCS (Lonza, Basel, Switzerland) and Myocyte basal medium (Pan Bio-Tech, Aidenbach, Germany) respectively. Cells were used until passage 5.

### Shock wave therapy

Electrohydraulic generated shockwave treatment was applied as described previously^15^. The Orthogold 180 device with applicator CE50 (MTS Europe GmbH, Konstanz, Germany) was used for all treatments. Respectively cells or animals were treated with 300 impulses with an energy flux density of 0.01, 0.07, 0.15 and 0.27 mJ/mm^2^ at frequencies ranging from 1 to 5 Hz. Common ultrasonic gel Skintact (Leonhard Lang, Innsbruck, Austria) was used for coupling.

### Necrosis assay

Thermo Scientific Pierce LDH Cytotoxicity Assay Kit (Thermo Scientific Waltham, MA) was used for quantitatively measure for cellular cytotoxicity and cytolysis. HUVECs, fibroblasts and cardio myocytes were seeded in T25 flasks, 96, 24, 12 and 6 well plates and cultivated in cell specific media (EGM (Lonza, Basel, Switzerland), DMEM (Pan Bio-Tech, Aidenbach, Germany), Myocyte basal medium (Promo Cell, Heidelberg, Germany)). 1 h after shockwave treatment LDH levels of supernatant were measured via Elisa reader, according to manufacture.

### Western blotting

Western blot for protein expression was performed as described previously^19^. HUVECs seeded in 6 well plates were processed 30 min, 1 h, 4 h and 6 h after shockwave treatment. The blots were probed with monoclonal rabbit anti-pAKT, monoclonal rabbit anti-AKT, monoclonal mouse anti-pERK (all Cell Signaling Technology, Cambridge, UK), polyclonal rabbit anti-ERK (Santa Cruz Biotechnology, Dallas, US, MA) and mouse β-actin (Sigma Aldrich, St. Louis, MO) antibody.

### Proliferation

24 h after shockwave treatment HUVECs, fibroblasts and cardio myocytes were fixed in 4% paraformaldehyde. Subsequently, DAPI (Abcam, Cambridge, UK) was used for nuclear counterstaining. Cells were examined using AxioVision Rel.4.8 software (Carl Zeiss, Oberkochen, Germany) and quantified with Image J (NIH, Bethesda, MD, USA)^30^.

### Tube formation assay

96 well plates were coated with matrigel (Corning, Munich, Germany) and incubated at 37 °C for 1 h. HUVECs were seeded and SWT was performed subsequently. Cells were treated with 10 mM SU1498 (Calbiochem, Santiago, Ca) for VEGF R2 inhibition. Pictures of tubes were acquired 5 h after seeding using AxioVision Rel.4.8 software (Carl Zeiss, Oberkochen, Germany) and quantified with Image J (NIH, Bethesda, MD, USA)^30^.

### qPCR

As reported previously total RNA was extracted from cells using the Monarch Total RNA Miniprep Kit (New England Bio Labs, Ipswich, MA) according to manufacturer`s instructions^19^. Briefly, real-time reverse transcription-polymerase chain reaction (qPCR) for gene expression analysis was performed with the ABI PRISM 7500 Sequence Detection System (Applied Biosystems, Life Technologies, Carlsbad, CA, USA). For performing the qPCR reaction a final volume of 14 µl containing 4 µl cDNA, 6 µl Luna Master Mix (New England Bio Labs, Ipswich, MA), 10 µm of Primer and 1,8 µl nuclease free water was used. Primers were designed using Primer Express Software (Applied Biosystems, Foster City, CA) and are listed below. The amplification consisted of a two-step PCR (40 cycles; 1 min denaturation step 1 at 95 °C for 15 sec annealing/extension step at 60 °C for 30 sec). Specific gene expression was normalized to the housekeeping gene GAPDH given by the formula 2 − Δ*C*t. The result for the relative gene expression was calculated by the 2-DDCt method. The mean *C*t values were calculated from double determinations and samples were considered negative if the *C*t values exceeded 40.

**Table:** 

	forward	reverse
VEGF human	gcctccgaaaccatgaactttc	caccacttcgtgatgattctgc
VEGF mouse	accctggctttactgctgtac	tcgctggtagacatccatgaac
VEGFR2 (KDR) mouse	tgatactggagcctacaagtgc	tgatgtacacgatgccatgc

### Animal experiments

Experiments were approved by the Austria animal care and use committee and were conform to the “Guide for the Care and Use of Laboratory Animals” published by the US National Institutes of Health (NIH Publication No. 85–23, 1996, revised 2011; available from: www.nap.edu/catalog/5410.html. Hind limb ischemia was induced as described previously^18^. Briefly, 12–15 weeks old male C57/BL6 mice (Charles River, Sulzfeld, Germany) were administered to anesthesia via an intraperitoneal injection of ketamine hydrochloride (Ketanest, Graeub, Switzerland, 80 mg/kg body weight) and xylazine hydrochloride (Xylasol, aniMedica, Germany, 5 mg/kg body weight). Popliteal artery and femoral artery proximal to the branching into saphenous were ligated with 7–0 polypropylene sutures (Ethicon, USA) and femoral artery was excited subsequently. Limb perfusion was measured using a laser Doppler perfusion image analyser (Moor Instruments, USA). Therefore, animals were kept on a 37 °C tempered heating plate. Limb perfusion was calculated as ration of left (operated, ischemic limb) to right (not operated, non-ischemic limb). For elevation of necrosis upon SWT in vivo, mice were administered to anesthesia as described above. Limbs were treated with SWT subsequently. 15 min after SW application skeletal muscle was harvested for histological processing.

### Immunofluorescence staining

Immunofluorescence staining was performed as described previously to analyze number of vessels^19^. Histological sections of the heart were incubated with monoclonal rat anti-CD31 (Dianova, Hamburg, Germany) or rabbit polyclonal anti-alpha smooth muscle actin antibodies (Abcam,Cambridge, UK) over night at 4 °C. For WGA staining histological sections were incubated at room temperature with WGA (Invitrogen, Carlsbad, CA) for 15 min. Five areas per sample were analyzed. Sections were examined with a Zeiss Axioplan 2 (Zeiss, Oberkochen, Germany) and a Leica SP5 confocal microscope (Leica, Wetzlar, Germany). Images were analyzed using ImageJ software (NIH, Bethesda, MD, USA) and processed with Adobe Photoshop CS5.1 for Mac (Adobe Systems Inc., San Jose, CA, USA)^30^.

#### Statistic

Graphs are presented as dot plots. Experiments illustrating a time course or dose response are represented as line graphs. All results are expressed as mean + SEM. Statistical comparisons between two groups were performed by Student’s t. Multiple groups were analyzed by one-way ANOVA with Tukey post hoc analysis to determine statistical significance. Probability values < 0.05 were considered statistically significant.

## Supplementary Information

Supplementary Information 1.

## Data Availability

The datasets generated during and/or analysed during the current study are available from the corresponding author on reasonable request.

## Abbreviations

HUVEC: Human umbilical vein endothelial cells
qPCR: Real time polymerase chain reaction
LDPI: Laser Doppler perfusion imaging
LV: Left ventricle
SW: Shock wave
SWT: Shock wave therapy
VEGF: Vascular endothelial growth factor
VEGFR2: Vascular endothelial growth factor receptor 2
IHD: Ischemic heart disease
