# Supplementary material for: Defining a therapeutic range for regeneration of ischemic myocardium via shock waves
Source: Sci Rep. 2021 Jan 11;11:409. doi: 10.1038/s41598-020-79776-z (PMC7801389; doi:10.1038/s41598-020-79776-z)

**Defining a therapeutic range for**

**regeneration of ischemic myocardium via shock waves**

Leo Pölzl, MD* ^a, b^, Felix Nägele, MD* ^a^, Jakob Hirsch, MD ^a^, Michael Graber, MD ^a^, Daniela Lobenwein, MD ^a, b^, Elke Kirchmair ^a^, Rosalie Huber ^a^, Christian Dorfmüller ^c^, Sophia Lechner ^a^, Georg Schäfer ^d^, Martin Hermann^e^, Helga Fritsch, MD ^b^, Ivan Tancevski, MD^f^, Michael Grimm, MD ^a^, Johannes S. Holfeld, MD ^a^, Can Gollmann-Tepeköylü, MD PhD ^a^.

*^a^ Department of Cardiac Surgery, Medical University of Innsbruck, Austria*

*^b^ Institute of Clinical and Functional Anatomy, Innsbruck Medical University, Austria*

*^c^ Heart Regeneration Technologies GmbH, Innsbruck, Austria*

*^d^ Department of Pathology, Medical University of Innsbruck, Austria*

*^e^ Department of Anesthesiology, Medical University of Innsbruck, Innsbruck, Austria*

*^f^ Department of Internal Medicine II, Infectious Diseases, Pneumology, Rheumatology, Medical University of Innsbruck, Innsbruck, Austria*

* Authors contributed equally

**Supplemental Figure:**

Full length blots of **a** ERK, **b** AKT, **c** pAKT, **d** pERK and loading control **e,f** via GAPDH staining.


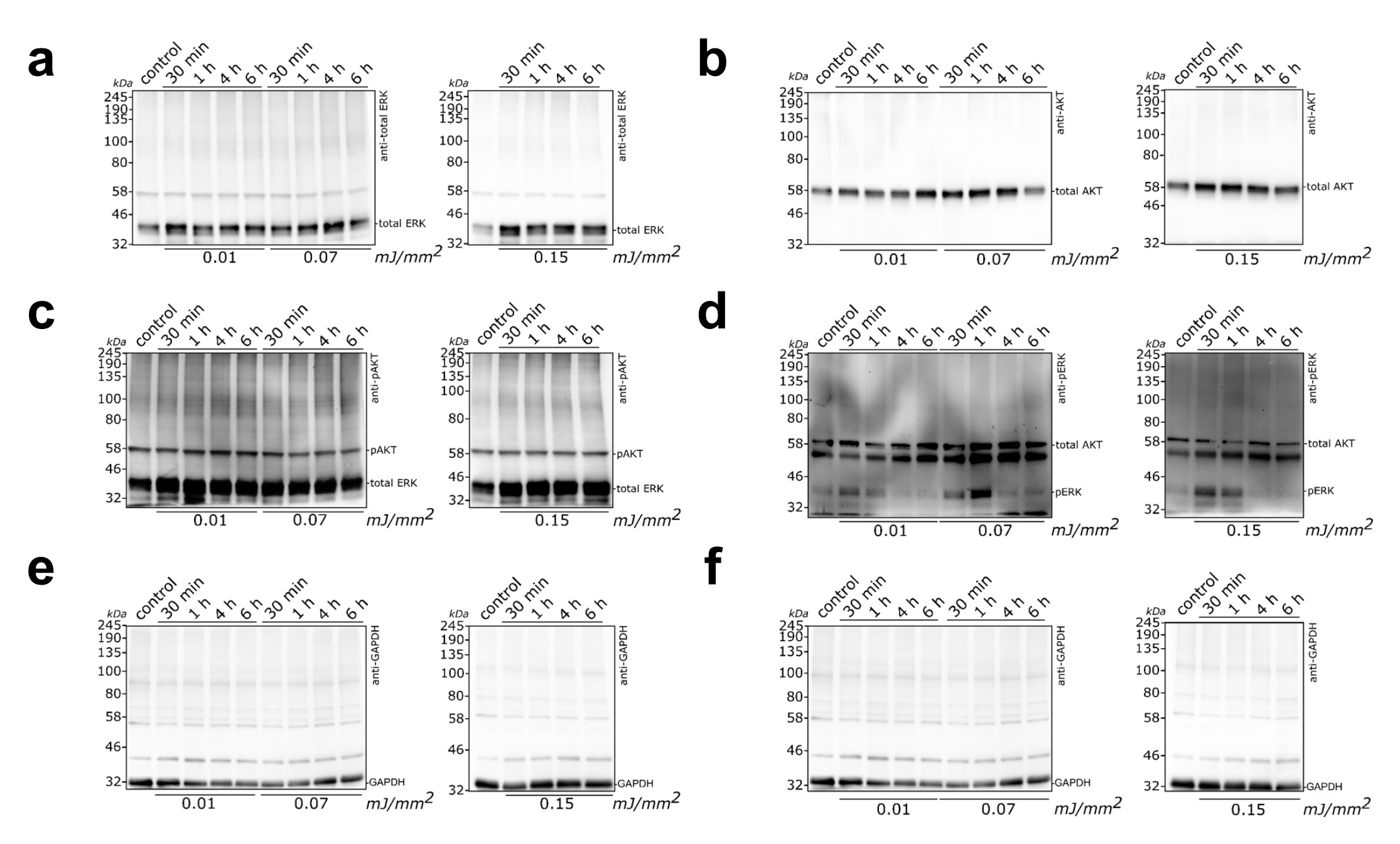

Supplement: Supplementary file 1 — Supplementary Information 1. [file 41598_2020_79776_MOESM1_ESM.docx]
